# Uncoupling Meat From Animal Slaughter and Its Impacts on Human-Animal Relationships

**DOI:** 10.3389/fpsyg.2020.01824

**Published:** 2020-08-04

**Authors:** Marina Sucha Heidemann, Carla Forte Maiolino Molento, Germano Glufk Reis, Clive Julian Christie Phillips

**Affiliations:** ^1^Animal Welfare Laboratory, Federal University of Parana, Curitiba, Brazil; ^2^School of Business Administration, Federal University of Parana, Curitiba, Brazil; ^3^Centre for Animal Welfare and Ethics, Faculty of Science, The University of Queensland – Gatton Campus, Gatton, QLD, Australia

**Keywords:** animal protection, animal suffering, cell-based meat, second domestication, human-animal relationship

## Abstract

Slaughter sets the debate about what is acceptable to do to animals at an extremely low bar. Recently, there has been considerable investment in developing cell-based meat, an alternative meat production process that does not require the raising and slaughtering of animals, instead using muscle cells cultivated in a bioreactor. We discuss the animal ethics impacts of cell-based and plant-based meat on human-animal interactions from animal welfare and rights perspectives, focusing on industrial meat production scenarios. Our hypothesis is that the insertion of cell-based meat in the global meat market may alleviate farm animal suffering and potentially restore resources for wild fauna. We employed a conservative estimation of the cell-based meat contribution to the global meat market in the year 2040 to analyze the consequences for human-animal relationships for both wild animals and farmed domesticated animals. We discuss possible effects of an animal cell domestication process, previously described as the second domestication, on human-animal relationships. We consider its potential to reduce the impact of human demographic changes and land use on animal life, in particular whether there would be increased biomass availability and free land for wild animals. We anticipate a major reduction in animal suffering due to the decrease in the number of individual animals involved in food production, which justifies the adoption of cell-based meat from a utilitarian perspective. For the conventional animal food production that remains, further consideration is needed to understand which systems, either high or low welfare, will be retained and the impact of the innovation on the average farm animal welfare. Additionally, it seems likely that there will be less acceptance of the necessity of animal suffering in farming systems when meat production is uncoupled from animal raising and slaughter, supported by a deontological perspective of animal ethics. Consequent to this is anticipated the mitigation of relevant barriers to animal protection and to the recognition of animals as subjects by legislation. Thus, the development of the alternative meats may be related to a significant change in our relationship with non-human animals, with greater benefits than the *prima facie* effects on farm animals.

## Introduction

Ever since humans first domesticated animals for the production of food, our manipulation of animals for the process has been expanding in scope. Darwin (1861) recognized our growing intervention in animal form and function in his Origin of Species: “Man selects only for his own good,” living as he did in an era and a country in which selective breeding was becoming widely used in agriculture. The next major event in selective breeding came with artificial insemination, allowing the so-called superior males to fertilize millions of females; then, embryo transfer, allowing the so-called superior female genes to be propagated more widely than through natural births; and, finally, or so we thought, cloning, perfecting the opportunity to perpetuate or even immortalize the genes of just one superior individual. However, over the last few decades, a technique for bypassing the animal altogether to produce meat has been in development, by growing muscle cells *in vitro*, which brings a different set of ethical questions and stances.

The main prompt for the development of these more efficient ways of producing meat is that the human population is expected to grow to 9.1 billion by the year 2050, which coupled with increased affluence that supports greater expenditure on food, requires annual meat production to raise substantially to 470 million tones (FAO, 2012). The need to alleviate food shortages and poverty suggests further intensification of animal production systems (FAO, 2018a), which is often associated with poor animal welfare (Bessei, 2006; Stafford and Gregory, 2008; Grandin, 2018). However, even with the development of incremental technologies for the intensification of production, the necessary gain in future meat production from agriculture may not be achieved (FAO, 2012). In addition, 48 authors from relevant institutions at national and international levels have signed the statement that “future technologies and systemic innovation are critical for the profound transformation the food system needs” (Herrero et al., 2020). Therefore, disruption of the conventional meat systems seems fundamental. Responses to this situation are under full consideration, as recently there have been much effort and investment in developing animal cell-based and plant-based meat alternatives. Both may potentially uncouple meat from slaughter, although each one faces important challenges, as for example, the fact that plant-based alternatives are not exactly meat and that cell-based options are not yet fully free of animal-derived ingredients. However, technology advances may bring the attributes of plant-based substitutes closer to those of conventional meat, as well as solutions for animal-free cell growth media.

Beyond the animal ethics benefits, additional advantages of replacing conventional meat for slaughter-free alternatives are straightforward: gains in environmental aspects, food security, public health, and food safety stand as the most clear-cut benefits, out of a long list of possible advantages (Gasteratos, 2019). Both plant-based and cell-based meat substitutes require less resource input per kilogram of product, as can be inferred from the impressive gains in carrying capacity, i.e., the number of people that could be fed from an agricultural land base, with changes from omnivorous diets to vegetarian or vegan diets (Peters et al., 2016), and from comparative estimates on cell-based meat production for environmental resource use (Tuomisto and Teixeira de Mattos, 2011; Röös et al., 2017). The overall environmental gains of diminishing conventional meat are also evident as the negative effects of the lowest-impact animal products typically exceed those of vegetable substitutes (Poore and Nemecek, 2018). In addition, the production of cell-based meat in closed bioreactors is expected to be sturdier in terms of climate, as compared to conventional meat, improving food security, which accordingly is one of the drivers for its development (Warner, 2019). The closed bioreactor environments may also contribute to a reduction in antibiotic use during meat production processes, which is a significant problem in conventional meat production due to the development of antibiotic resistance (Aires-de-Sousa, 2017). In relation to nutrition security, an important consideration is that meat is a protein source of the highest biological value, second only to egg and milk proteins (Hoffman and Falvo, 2004), while plant-based substitutes require more research and efforts to approach conventional meat amino acid value as human food. Cell-based meat offers additional advantages in comparison to conventional meat, as its proteins are coded by animal cell DNA, which tends to maintain conventional meat amino acid profile, and its final overall composition may be customized in a tailored way, such as low cholesterol risk by using mostly poly and monounsaturated fatty acids, for example. Finally, both meat alternatives offer virtually zero risk of zoonotic diseases, as pathogens are intrinsically absent in the production process. Thus, innovative meat products tend to significantly reduce human suffering and financial costs associated to both prevention and treatment actions required by the conventional meat chain regarding bacterial diseases, such as those caused by *Salmonella*, *Escherichia coli* O157:H7, methicillin resistant *Staphylococcus aureus* (MRSA), and bovine tuberculosis. In addition, dangerous virus mutations, such as the new subtypes H5N1 and H7N9 of Type A influenza virus, popularly known as bird flu, the subtype H1N1, known as swine flu, and the recent SARS-CoV-2, the coronavirus causing Covid-19, would be impossible with the consumption of alternative meats. This is a major benefit as these diseases are causing major human mortality and current control measures are seriously disrupting human society.

Unlike the classic plant-based substitutes for meat, which used whole vegetable ingredients such as peas and other beans, many of the new plant-based meat analogs are structurally similar to meat (Joshi and Kumar, 2015), as they are molecularly constructed. Even though they differ in composition, these substitutes preserve certain properties and sensory attributes of meat, such as texture and flavor (Dekkers et al., 2018). The process of formulating these products includes a comprehensive molecular analysis of plant proteins in search of compounds that simulate animal meat (Lagally et al., 2017). Another emerging technology is the use of genetically modified bacteria and yeasts to generate organic molecules for the production of gelatin, collagen, milk, egg white, etc. through fermentation (Stephens et al., 2018). To produce cell-based meat, the same fundamentals of tissue engineering technology that have been perfected in the last few decades are used, including the proliferation and differentiation of specific stem cells for each tissue required to match meat compounds, such as muscle and fat (Datar and Betti, 2010; Post, 2012; Ben-Arye and Levenberg, 2019; Zhang et al., 2020). Thus, the resultant meat is potentially the same as that from farm animals but made through a slaughter-free process. Start-up companies working with cell-based technology may be considered disruptive as they use different and potentially fewer resources to develop an improved method of producing meat, which in turn may potentially transform the food chain. Thus, a new set of capabilities beyond the evident biotechnological knowledge required will characterize the cell-based meat global value chain (Reis et al., 2020). Furthermore, cell-based meat may change historical concepts, perceptions, and practices, in the context of human-animal relationships. The domestication of animals as sources of food over the last 10,000 years has changed human society and the role animals play in it. Recently, with the beginning of cell-based technology, a new domain is possible: the domestication of cells rather than animals (Shapiro, 2018; Tubb and Seba, 2019). Similar to the events of the first domestication, cells rather than animals may in future be genetically selected, raised, and fed an optimal diet.

The development of cell-based meat and other cellular agriculture techniques may therefore be considered “disruptive innovations,” i.e., likely to remodel the different sectors of the industry or services (Christensen et al., 2015). These technologies also encompass the three attributes that define radical innovations (Dahlin and Behrens, 2005): uniqueness, novelty, and likely to influence future innovations. They employ unique and novel processes for producing meat, i.e., processes which are different from previous and current ones and may redefine the future technology used in the meat and agribusiness chains as a whole. In relation to animal products, a disruption may be dependent on whether consumers have attitudes that lead them to search for aspects beyond quality and price to include ethical aspects, regarding animal welfare and the environmental impact of meat, for example (Goddard, 2019). This occurs mostly in the early stages of the disruption, since in the medium-term product quality likely improves and acceptance tends to increase, especially if prices decline, which will almost certainly occur as new technologies are developed. If such a disruption to our food chain eventuates, a change in human-animal relationships is likely to occur, as for the first-time, it will be possible to challenge the concept of necessary animal suffering and killing without compromising meat consumption. Pressure from the animal production industry has been limiting the farm animal protection laws (Schwartz, 2020), which commonly prohibit only unnecessary suffering of farm animals. This is designed to shield harmful practices in animal production systems from inclusion in the list of crimes against animals, or even more deeply, from the very recognition of farm animal suffering and abuse. Most of all, the acceptance of the slaughtering of animals for food sets any debate about what is acceptable to do to animals at an extremely low bar. Many forms of animal abuse that are associated with legitimate goals, such as scientific experimentation and food production, are sustained by institutions with important social credibility. Therefore, it seems that society will allow certain contexts of animal cruelty without question (Flynn, 2012), because a genuine benefit from the practices is perceived.

Accordingly, cruelty to animals is often legally focused on the avoidance of unnecessary suffering (Radford, 2001), which is defined as avoidable and purposefully caused. This is considered to infringe moral principles (Hurnik and Lehman, 1982). In addition, there are many different interpretations of animal suffering, depending on the country, culture, and animal species in question (Lundmark et al., 2014), including which animal species are considered edible (Herzog and Foster, 2010; Joy, 2011). Although farm procedures causing pain and distress imply suffering, most policymakers interpret them as necessary, e.g., beak trimming of turkeys, laying hens, and castration of piglets (Lundmark et al., 2014), as they prevent behavior problems in high density stocking and consequently economic losses. Thus, legislation regarding animal suffering is contradictory due to the inconsistency in policymaker conclusions (Lundmark et al., 2018). This is one example of ways through which traditional meat production axioms tend to naturalize or even to extol animal suffering and killing; this normalization process may generalize and is likely not restricted to those animals used in food production activities. However, animal ethics is gaining unprecedented recognition in current western societies. The dilemma about how we use animals, and if we “use” them at all has become a major ontological, epistemological, moral, and political force, and it may be that a profound anthropological shift is underway (Burgat, 2015). It is our view that a basic hindrance for this anthropological shift is the persistent motivation to eat meat. Thus, the development of a system that makes meat production possible without animal suffering is likely to cause profound changes in the human-animal relationships.

In this paper, we discuss the ethical impacts of alternatives to conventional meat on human-animal interactions from an animal point of view, focusing on industrial meat production scenarios. Our hypothesis was that the insertion of plant-based and cell-based meats in the global meat market may alleviate farm animal suffering and partly restore habitat for wild native fauna, in addition to creating new possibilities for animal ethics and protection, as it relieves the need to accommodate the necessary animal suffering and killing that accompany modern animal production practices.

## Materials and Methods

### Scenario Forecasting

The evidence suggest that alternative meat production methods will become a reality, leaving little room to speculate whether they will hold an important position in the food industry, rather only questions regarding time frame. The market share of plant-based meat substitutes has consistently increased since it was launched, with data from the United States showing that retail sales of plant-based foods grew 11.4% in 2019, within a context of overall food retail growth of 2.2% (Plant Based Foods Association, 2018), and more recently, the Covid pandemic outbreak resulted in a further increase in sales of plant-based meat substitutes, likely caused by perceived high product safety regarding zoonotic diseases and the many difficulties related to Covid outbreaks within slaughterhouses. Regarding cell-based meat, even though it is not yet on the market, the increasing number of start-ups with robust and increasing investments dedicated to its development constitutes a sign of accelerated development. In the United States, the Food and Drug Administration (FDA) and the United States Department of Agriculture (USDA) have recently engaged in conversations regarding cell-based meat labeling and regulation, essentially to align on a joint regulatory framework between the two agencies (Congressional Research Service, 2018; USDA, 2019a). In Europe, newly developed foods, such as cultivated meat, are regulated under the Novel Food Regulation supported by the European Food Safety Agency (EFSA), with labeling regulations from Food Information to Consumers (FIC; Froggatt and Wellesley, 2019).

These movements by such institutions seem powerful indications of the relevance of this new industry. However, there are uncertainties as to the exact proportions of total meat market to be substituted, which are challenging for scenario forecasting. For instance, although recent research has shown that cell‐ and plant-based meat substitutes may be accepted or at least tried by consumers in a diversity of countries like Brazil, Germany, Italy, India, China, and the United States (Bryant et al., 2019; Mancini and Antonioli, 2019; Valente et al., 2019; Weinrich et al., 2019), some of those products do not exist so far (e.g., cell-based meat products), and more nuanced insights into the cultural and social barriers for introducing food innovation are still needed (Herrero et al., 2020), as they can challenge an exclusively technical understanding of dietary changes (Noack and Pouw, 2015). Thus, even though the need for a profound transformation of the food systems is recognized (Herrero et al., 2020), projections must be cautiously interpreted.

In line with the prevailing uncertainties, we employed a conservative estimation, one that is both cautious and moderate, of the cell-based meat contribution to the global meat market in the year 2040, to analyze its potential consequences for animal welfare and the human-animal relationships. As a recent scientific development, cell-based meat projections are scant in scientific literature; thus, our discussion is based on the prospective agribusiness disruption in global industry and economy for 2020–2030 and 2040 presented in reports by Tubb and Seba (2019), an independent team of technology, finance, and market experts, and the global consultancy group AT Kearney (Gerhardt et al., 2019), the only available documents with such projections. Due to the limitations in knowledge, at this point, a major emphasis on scale rather than absolute numbers seems warranted, thus reducing expectations of precision and error risks. Knowledge is limited and is curbing cell-based meat development, in terms of intrinsic factors such as animal-free culture medium ingredients, scaling-up challenges, and final product characteristics. A variety of extrinsic factors may additionally affect the development rate of meat substitutes and are difficult to predict. Examples of external relevant factors are climate change, water shortages, outbreaks of food-borne diseases, as well as the geographical distribution of these putative events, which may differently stress either a faster or a slower development for each plant‐ and cell-based meat alternatives. Furthermore, we highlight again that as potential consumers worldwide have socially engrained relationships to food (Herrero et al., 2020), expressed as established local habits and traditions, the acceptance of meat substitutes may not be straightforward. Considering all the complexities, however, it seems clear that a major disruptive change is on the horizon, which warrants forecasting efforts from a variety of perspectives. We are specifically interested in understanding how it will change human-animal interactions. For this, a preliminary scenario assumption in terms of the magnitude of the changes is required.

Tubb and Seba (2019) used data from the United States to calculate frameworks and information from The Good Food Institute, a non-governmental organization that supports cell-based studies, to reference their analysis of cell-based products. The report is focused on cattle; however, it includes some information on other food animal production systems, as well as information on clothing and cosmetics. It suggests that the ability of cell-based products to transpose the conventional systems is high, starting with ground meat and reaching afterward into the integral muscle tissue markets, such as steaks. Precision fermentation of genetic modified microorganisms may also be utilized to produce specific proteins needed for culture media and to provide animal products other than meat, such as milk and eggs. It is estimated that in the year 2030, 30% of the conventional beef in the United States will be substituted by cell-based meat, and the cost will be substantially less than that of conventional meat. Independently, Gerhardt et al. (2019) combined opinions from experts in the global agriculture, food, and meat industries to conceptualize what alternative sources of meat may be in use in the year 2040. They estimated that cell-based meat will represent 35% of the global meat chain in the year 2040 and plant-based meat another 25%. Thus, conventional meat may be reduced to 40% of total meat production by the year 2040.

For this paper, we used the statistics of Gerhardt et al. (2019) due to the report’s worldwide analysis and more conservative perspective in terms of both percentages and time frame, comparing its 2040 scenario to the 2030 one considered in the Tubb and Seba (2019). Subsequently, we applied the expected reduction of 60% in traditional animal production for the year 2040, including 35% of cell-based and 25% of plant-based meat replacements (Gerhardt et al., 2019), to study the direct impact on number of animals involved and biomass distribution across terrestrial vertebrate animals. Our analyses considered the major production chains involving cattle, pigs, and chickens. The 60% was chosen as the most conservative prediction from an extremely limited choice between two publications and, as such, its interpretation is subject to the background consideration of the aforementioned relevant intrinsic and extrinsic factors at play. More extreme percentage substitutions of conventional meat may be considered as potential lower and upper limits. If technological challenges for cell-based meat development prove too challenging, the respective 35% predicted market share will not be achieved within the considered time frame, which would leave the overall substitution by the year 2040 at around the 25% predicted for plant-based alternatives, assuming that there would not be a compensatory emphasis on plant-based developments. Another powerful restrictive condition is the launching of cell-based meat as an animal friendly product before the complete substitution of animal-derived ingredients in the cell culture media. If this occurs without due transparency to consumers, the consequences could include a strong backlash, with the attachment of a strong negative image to any future cell-based meat product. At the other extreme, much higher percentage substitutions may be achieved if technological breakthroughs present themselves before the year 2040 and if stricter animal protection laws come into effect as a consequence. Some restrictions to harmful animal use when alternatives exist are currently in place in many countries in other contexts, such as the use of animals in science. The same rationale may be put in place, considering the raising and killing of animals to produce meat, which would lead to levels of substitution closer to 100%, aided by legal restrictions on animal use, which are unlikely to be enacted simultaneously in different countries.

### Direct Impacts of Alternative Meats on the Environment and Vertebrate Terrestrial Animal Biomass Distribution

We considered the impacts of the replacement of conventional meat sources with 35% of cell-based and 25% of plant-based meats by the year 2040 on the environment, addressing land, water and energy use, as well as for the vertebrate terrestrial animal biomass. Then, we studied biomass impact, considering that biomass is the metric used to quantify carbon usage by different organisms. Based on the estimation of biomass distribution by Bar-On et al. (2018), which measures biomass in gigatons of carbon (1Gt C = 10^15^ g of carbon), we applied the estimated 60% (35 and 25%) reduction of livestock biomass by the year 2040 (Gerhardt et al., 2019), to estimate the potential biomass release.

### Direct Impact of Meat Alternatives on Farm Animal Welfare

The estimation of the reduction in the number of individual farm animals as a consequence of the introduction of 35% of cell-based and 25% of plant-based meats was based on the predicted global beef, pork, and chicken meat production for the year 2040 and the current number of cattle, pigs, and chickens. Even though the highest number of individual vertebrate animals involved in food production is that of fish species, which supports the need for urgent action regarding their welfare, data on an individual animal basis are very difficult to estimate, and they were not included in this exercise. Fish are consumed in part because the meat is believed to confer health benefits, and as such, the opportunities to value-add by improving the health giving credentials of the meat are considered to be less than for terrestrial animals and, therefore, less likely to be a target for replacement.

First, we calculated the production for these chains using values from the years 2017, 2019, and 2020 (Table 1), which we considered represented current production. Then, we calculated the average of the published prospective world meat production for the years of 2027, 2030, and 2050, to estimate animal meat production for the year 2040 (Table 1). In this exercise, the potential dynamics of the interplay among the three terrestrial meat production chains across the next decade, namely cattle, pigs, and chickens, were considered stable, to reduce complexity in the calculations, even though some changes in proportions may occur, as chicken meat production is growing at a faster rate than cattle and pig production. However, we assumed that this dynamic character may not sufficiently change numbers to invalidate our conclusions.

**Table 1:** Meat production estimation, in million tones for beef, pork, and chicken.

Production chain	Data source			Average
	FAO	OECD	USDA	
Beef	70.8 (2017)^1^	72.7 (2019)^2^	61.9 (2020)^3^	68.5
Pork	118.7 (2017)^1^	121.8 (2019)^2^	95.2 (2020)^3^	111.9
Chicken	120.5 (2017)^1^	125.3 (2019)^2^	103.5 (2020)^3^	116.4
Total	376.0 (2030)^4^ 470.0 (2050)^5^	367.0 (2027)^2^	—	404.3 (2040)^6^

1 FAO, 2018b;

2 OECD, 2018b;

3 USDA, 2019b;

4 FAO, 2003;

5 FAO, 2012;

6 Our estimation.

Afterward, we calculated the average stock number for each species using published data from years 2017 and 2019 (Table 2). Two of the references cited did not present the quantities of pigs (FAO, 2019) and chickens (USDA, 2019b); therefore, we left this data out of the calculation. Also, for cattle, most of the references present data from both the beef and dairy industries; hence, we selected the data from USDA (2019b), which referred only to beef cattle. Later, we calculated the percentage of production growth from the year 2020 to 2040 and applied this number to each previous animal individual population. Finally, we calculated the reduction of individuals in each animal species for the future, following the estimation of 60% by Gerhardt et al. (2019; Table 2). The decrease in the number of individual animals involved in meat production was considered a straightforward gain in animal welfare and in animal ethics. The animal welfare gains refer to the reduction of total animal suffering, composed of the summation of individual afflictions, as animals involved in intensive production systems suffer from severe space and consequent behavioral restrictions, health problems resultant from artificial selection for production traits, and submission to painful procedures and stressful management events, such as transport and slaughter (Harrison, 1964; Webster, 2005; Broom and Fraser, 2015). Gains in animal ethics include all the welfare gains, in addition to the proportional absence of breaches in animal integrity and dignity, which are inherent to the killing of each sentient individual. In other words, the killing of animals is an important moral issue because of the suffering involved (Višak and Garner, 2016).

**Table 2:** Estimation of number of individual animals, in billions, based on cattle, pig and chicken stock number published in four sources in 2017-2019, and in 2027-2050, after multiplying by the percentage increase in production growth calculated from total annual meat production per species as per Table 1.

Source	Animal species			
		Cattle	Pigs	Chickens
FAOSTAT (n.d.)	2017	—	1.41	27.82
STATISTA (n.d.)	2017	—	0.78	22.85
FAO, 2019	2017	—	—	18.30
USDA, 2019b	2019	0.99	0.77	—
Calculated average	2020	0.99	0.98	22.99
Forecast based on estimation for 2040^1^$\%=\frac{404.3\;x\;100}{296.8}$	2040	1.34	1.34	31.32
Forecast based on 60% substitution by meat alternatives^2^	2040	0.54	0.54	12.53

Production levels after applying the anticipated reduction of Gerhardt et al. (2019) are also provided.

1 As calculated by (404.3 × 100)/296.8 (see Table 1) and weighed for the proportion of each animal species;

2 Gerhardt et al. (2019).

Finally, we envisioned three possibilities for the individual animals that will remain involved in production in the year 2040: (A) the welfare and number of farm animals if conventional meat production was to remain the sole system in the year 2040; (B) the average welfare and number of the remaining farm animals if conventional meats were to compete with cell‐ and plant-based meats for low-priced products; and (C) the average welfare and number of the remaining production animals if conventional meats were to compete with cell‐ and plant-based meats for high-priced products. Scenario A is fictitious and presented only for comparison, as in 2020, plant-based alternatives to meat products can already be purchased in many supermarkets, as well as restaurants, including major fast-food chains, such as A&W, Burger King, Kentucky Fried Chicken, and Subway.

### Indirect Impacts of Alternative Meats on the Human-Animal Relationship

The impact of increasing markets for cell-based and plant-based meats on the human-animal relationships was analyzed using two complementary rationales. The first is related to a reduction in the negative impact of conventional meat production on global animal welfare, particularly in intensive raising conditions and during slaughter, which is avoided every time conventional meat is replaced by an alternative product. The second rationale is that, due to the extinction of the meat paradox, there may be fewer people who are desensitized toward animal suffering. The meat paradox is defined by Loughnan et al. (2014) as the simultaneous emotion related to the fact that people tend to dislike hurting animals and, at the same time, to like eating meat.

### Results and Discussion

According to our analysis of the reduction in the number of animals used in the production for the year 2040, we discuss the impacts of alternative meats on the environment and biomass distribution, on farm animal welfare, and on the human-animal relationships.

#### Environmental and Vertebrate Animal Biomass Consequences

Livestock production uses extensive areas of land and is responsible for the occupancy of 26% of the terrestrial land, as well as 33% of the total arable land, which is dedicated to crop production for animal feeding (Steinfeld et al., 2006). The expansion of grazing areas and crop planting to feed farm animals has been related to deforesting important ecosystems. For instance, 70% of the deforested area of the Amazon forest is occupied by pastures for grazing animals (Steinfeld et al., 2006). This decreases resources for wildlife (Steinfeld et al., 2006; Tuomisto and Teixeira de Mattos, 2011). According to studies of prospective high-volume cell-based meat production (Tuomisto and Teixeira de Mattos, 2011; Mattick et al., 2015), large amounts of land, up to 99% of that currently used, will be freed (Tuomisto and Teixeira de Mattos, 2011). The new system of producing meat will surpass the efficiency of land use even when compared to the intensive meat production involving pigs and chickens (Mattick, 2018). Since cell-based meat production will be conducted in bioreactors, it is likely that there will be major transformations in the industrial production landscapes, which are calculated to be much less dependent on land use. Therefore, some land space will be freed, and this may return to wildlife or be used for further expansion of the human population, or both. The latter seems unlikely as land availability does not appear to be a constraining factor on human population growth, with most growth occurring in the urban population (FAO, 1999).

Regarding water consumption, agriculture accounts for 92% of the human fresh water footprint and almost one-third of this relates to animal production (Gerbens-Leenes et al., 2013). Additionally, considering the continuous expansion of the livestock population for animal-derived products, any intensification of production may increase water use due to a greater dependence on concentrate feed (Mekonnen and Hoekstra, 2010). Tuomisto and Teixeira de Mattos (2011) estimated that there would be a reduction of 82–96% in water consumption for each kilogram of meat produced, comparing cell-based and conventional animal meat production systems. As with all estimations regarding cell-based meat, this number is dependent on assumptions, which are not yet all clear; however, the scale makes the estimations relevant, for both land and water use. Even if we consider some inaccuracy in the estimations, a major reduction seems probable. At the same time, as land and water use are likely to considerably decline, energy inputs may increase for cell-based meat production due to the greater demand for electricity by laboratories in all phases of the cultured meat production process (Tuomisto et al., 2014; Mattick et al., 2015). Hence, improvements in the efficiency of energy use, such as developing clean and renewable alternative sources of energy, will remain an important requirement. As an overall effect of the reduction in the number of individual animals used for meat production, some of the released natural resources will be needed for biomass production for energy generation.

**Figure 1:**
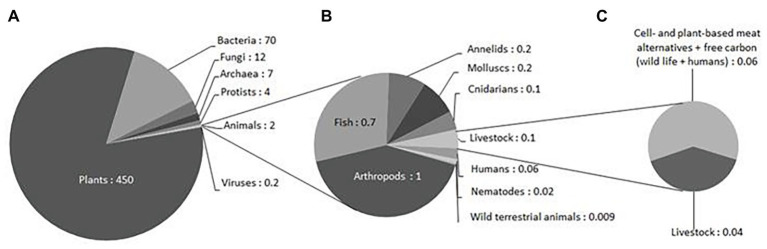
Biomass distribution for kingdoms **(A)**, animal groups **(B)**, as per Bar-On et al. (2018) and the analysis of the impact of a 60% reduction in slaughter-based meat production **(C)** (Gerhardt et al., 2019) on the availability of biomass (1 Gt of carbon = 1Gt C = 10^15^ g of carbon).

The biomass of carbon in livestock, concentrated in cattle and pigs, is much higher than that in wild mammals: ~0.1 Gt C, compared with 0.007 Gt C (Bar-On et al., 2018). That in domestic poultry, mostly chickens, is in turn greater than that in wild birds: 0.005 and 0.002 Gt C, respectively (Bar-On et al., 2018). Our assumption is that the reduction of 60% in the number of farm animals when cell-based meats and plant-based alternatives are developed may release 0.06 Gt of carbon biomass (Figure 1); this surplus is related to the increase in efficiency characteristic of the alternative forms of meat production. Additional studies describing the biomass requirement for alternative meats are required, since they may give a more precise idea of the carbon amount, which may be liberated, and thus available for either animal wildlife or expansion of the human population, or both. However, from the Figures presented here, it is apparent that today’s biomass available for wild terrestrial animals, at around 0.009 Gt C, would be greatly augmented by the reduction in the number of farm animals, which may release 0.06 Gt C by the year 2040. In other words, the amount of carbon released due to the reduction in the number of farm animals is 6.7 times the amount of carbon currently available for all wild terrestrial animals. Even considering that part of this freed carbon will be sequestered in the form of cell-based and plant-based meats, the possibilities for partially restoring wildlife biomass seem encouraging.

#### Impact on Animal Ethics and Welfare

Animal ethics is the branch of ethics that relates to human-animal relationships and how human ought to treat other animals. Conversely, animal welfare is based on empirical science, informing humans of the quality of an animal’s life, based on the extent of good and bad experiences that the animal is having, has had, or is expected to have (Phillips and Kluss, 2018). By definition, it is the state of an individual regarding its attempts to cope with its environment (Broom, 2011), and it is measurable by considering animal’s physiology and behavior. Animal cells, extracted from livestock for the purpose of generating cell-based meat, cannot be said to have rights, in the same way as animals, because such rights are based on animals’ interests (Beauchamp, 2011). However, the cells may be said to have their own needs, which give them maximum advantage. Animal rights protagonists may further argue that if animals have the right not to have their bodies or parts of their bodies used in biomedical research, because it challenges their body integrity, they may also have the right not to have their muscle cells extracted for cell-based meat production. However, from the perspective of the continuum of attitudes toward animal rights advocated by Beauchamp (2011), such views represent an attitude founded at the extreme end of the animal rights continuum, particularly if there are utilitarian benefits to the species or specific animals involved. Beauchamp (2011) suggests that rights only merit protection if the benefits accrue to the individual animals themselves, not the species; hence, the impact on the animal from whom the cells are extracted merits detailed consideration. In addition to extracted cells, fetal bovine serum is currently used to grow cell-based meat (Chauvet, 2018). This serum is an excellent source of nutrients and cell-growth factors, and it is collected from fetuses at abattoirs. During slaughter of the cow, the fetal heart is punctured to extract blood, and there is a concern that the fetuses may still be alive during the process, which may even be considered an advantage by some because it is possible to extract more blood if the heart is still beating (Phillips and Kluss, 2018); the blood thus collected is then processed for fetal serum production. Fortunately, it is realistic to expect a non-animal replacement for the fetal bovine serum in the near future (Chauvet, 2018). Fetal serum substitution is currently under development by adapting cells to chemically defined media, which are fully independent of animal-derived ingredients (Marigliani et al., 2019a). Fetal bovine serum is not the only animal ingredient used in cell culturing; a systematic review of 156 articles featuring 83 different cell culture methods identified the use of several animal-derived products from different species (Marigliani et al., 2019b). A major advancement in this issue came with the publication of the new Organisation for Economic Co-operation and Development (OECD) Guidance Document on Good *In Vitro* Method Practices (OECD, 2018a), discouraging the use of serum and presenting a list of serum-free media alternatives, including an animal-product-free media description. The challenges of offering meat that is really cruelty-free and that is also perceived to be so may likely be overcome by implementing technology for the use of culture media that is completely free of animal ingredients and by adopting strict transparency so not to risk a breakdown in consumer confidence.

A fundamental objection to the use of animal cells for the production of cell-based meat is that it promotes the concept that animals are a legitimate source of food, a view challenged by many animal rightists. Human cells could equally well be used to produce cell-based meat; however, they would be accepted by few consumers (Wilks and Phillips, 2017). Many surveys worldwide have demonstrated that most people would accept the use of animal cells in cell-based meat and would at least try the product (e.g., in the United States, Wilks and Phillips, 2017; in Brazil, Valente et al., 2019). The biggest impediments to its more permanent adoption are likely to be food neophobia, political conservatism, and a distrust of scientists (Wilks et al., 2019). A related concern, levied against the use of genetically modified animals, is that humans are “playing God and against Nature” (Savulescu, 2011). The concern derives both from a perceived attempt by humans to usurp the role of a higher being and also an overestimation of our ability to manage complex biological systems. The latter is related to people’s distrust of scientists, when it comes to their ability to create new food sources safely (Wilks et al., 2019). A further concern is the slippery slope argument (Savulescu, 2011) that assumes that innovations such as cell-based meat will ultimately lead to more damaging innovations that will seriously degrade human society, for example, creating cell-based meat based on humans. This concern may be challenged by the idea that each step in our manipulation of life on earth is checked in terms of its benefits for society as a whole. Without central control by government, human life would be “poor, nasty, brutish, and short” (Hobbes, 1651). However faulty this system may be, it is undeniable that human intervention has improved human life quality and quantity throughout many centuries. It is possible and urgent that human interventions care for other sentient beings and for the environment in a more solid and straightforward manner.

**Figure 2:**
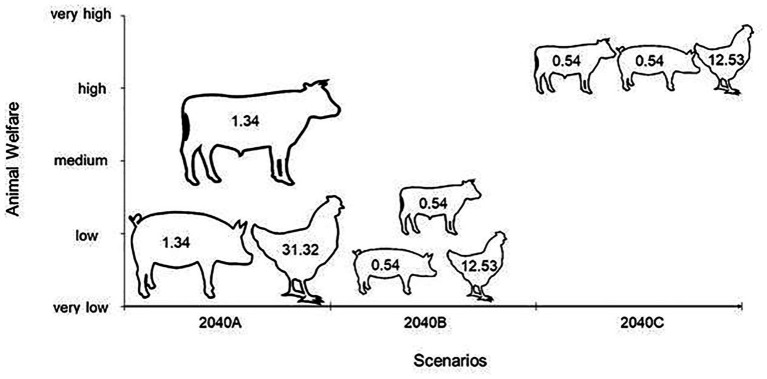
Number of individual animals in each degree of animal welfare, in billions, considering the estimated total number of cattle, pigs, and chickens in 2040, assuming that total global meat production will be reduced to 40% of its 2019 level, following the projected insertion of 35% of cell-based and 25% of plant-based meat production (Gerhardt et al., 2019).

Another concern is the detrimental impact that cell-based meat may have on existing livestock numbers worldwide. It has been assumed that cell-based meat would compete with high-value meats, not industrially produced low quality meats (Cole and Morgan, 2013). However, other possibilities must also be considered. In Figure 2, the number of individual animals involved in each of the three most relevant global meat chains is presented, and the scenarios B and C posit quite different responses of the animal production industries to the insertion of the alternatives to traditional meat in the global market. The validity of this ethical objection depends not so much on which scenario is correct, rather on the answer to the question of whether farm animals’ lives are worth living at all. The “life worth living” concept, which emerged from considerations of the quality of human lives (Yeates, 2017) has been developing from a motivational framework, in which it appeared in its infancy (Webster, 2016), to a more robust concept that can be used to measure, or at least estimate, animals’ quality of life (Mellor, 2016). If cell-based meat does compete with high-end meat products, appealing to the ethical consumer, these are likely to be derived from livestock with the best welfare, even considering the limited range of welfare for most farm animals. However, the market for inexpensive, mass produced meat has been growing at the expense of the quality product, and this market may well be one target of cell-based meat manufacturers, given that production costs are expected to decrease and to reach cost parity with conventional meat products in the next 5–10 years (Tubb and Seba, 2019). This mass-produced meat originates from intensive production systems, where it is debatable whether animal lives are worth living. Furthermore, diminishing the use of agricultural land for animal production will free up land, where wildlife may be allowed to flourish.

Pressure on wildlife habitat from expanding agricultural production is at least partly responsible for the novel zoonotic wildlife diseases that are emerging (Wilkinson et al., 2018). This substitution of farm animals by other forms of life may dramatically change the distribution of vertebrate animal life on earth (Figure 1). Few comparisons of farm and wild animal numbers exist, but in the case of birds, the global biomass of domestic poultry is three times that of wild birds, as described above (Bar-on et al., 2018). Similarly, the biomass of humans and livestock outweighs that of terrestrial wild vertebrates. As it is widely acknowledged that the welfare of farmed livestock is poor (Phillips, 2015), replacement with wildlife that is subjected to fewer anthropogenic pressures is morally justifiable, even desirable from a utilitarian standpoint. From a deontological standpoint, there are additional concerns about the short lives of farm animals, infringing Tom Regan’s concept of subject of a life (Beauchamp, 2011), the manipulation of their genetic inheritance as a species, and threats to their future existence caused by limitation of their biodiversity (Phillips, 2015), again suggesting that substitution with wildlife is desirable. There may be concerns that the welfare of wildlife, particularly of prey animals, is also compromised, but then Darwin (1861) had considerable insight: “we may console ourselves with the full belief that the war of nature is not incessant, that no fear is felt, that death is generally prompt, and that the vigorous, the healthy, and the happy survive and multiply.” Today, this statement may be recognized as somewhat romanticized; however, it seems relevant to acknowledge animal ethics gains from decreasing animal suffering, which is directly anthropogenic.

Scientific assessment of animal welfare has been the object of many scientific papers and has now been summarized in protocols. The most used protocols for the animal species represented in Figure 2 are the respective Welfare Quality protocols (Welfare Quality®, 2009a,b,c), and they include a variable number of specific measurable indicators for each of the four principles: good feeding, good housing, good health, and appropriate behavior. The measured levels for each indicator are composed of the degree of adherence to each principle, which in turn are integrated to calculate a final welfare level for the target situation. Recurrent animal welfare assessment has produced a relatively improved understanding of welfare status for the most common animal production systems. In general, giving livestock access to pasture improves most aspects of their welfare (Mee and Boyle, 2020) in contrast to increasing use of intensively confined systems employed for most of the pig and broiler chicken industrial farms. For this reason, in current practices involving most of the animal industry, it is possible to distinguish welfare levels of pastured cattle as relatively higher than those of indoor-raised pigs and chickens, as represented in Figures 2A,B. This approach simplifies complexities which are inherent to the many field variations that may be observed when assessment is performed and rather uses a concept of animal welfare potential of each system. However, it relies on our best assumptions of welfare, as per current knowledge. Although many scientific studies have proposed solutions to prevent animal welfare issues, they still persist and even major problems with simple solutions became normal in production systems (Grandin, 2018). The intensive systems of pig and chicken industrial production are often related to poor living conditions for the animals, such as high stocking densities and early growth diseases (Bessei, 2006), and even animal welfare certified systems may not present significant improvement for the animals (Souza et al., 2015; Reis and Molento, 2019). Therefore, even though there may also be issues related to the extensive production systems (Petherick, 2005), the intensification processes seem to intrinsically reduce the welfare of the animals. In addition, we have only considered straightforward conditions of animal raising and slaughtering and aberrant situations such as overseas live exports were not included; even though these situations are extremely relevant, their inclusion would have blurred the picture due to the level of details required. Thus, in Figure 2A, we have distributed cattle, pigs, and chickens according to their average animal welfare in industrial production systems described in a simplified but representative way, in terms of what happens to the greatest number of animals in each species, as well as the number of individuals predicted to be involved in the year 2040 if no alternative meats were to become significant in the global market.

Since plant-based and cell-based meat production strategies are virtually animal-free systems (Kadim et al., 2015), if the scale of the forecast turns out roughly correct, a substantial decrease in the number of animals involved in intensive raising practices and slaughter will occur, which will in turn significantly impact the total animal suffering. Even though animals may still be necessary for cell supply, the techniques available to induce cells to proliferate indefinitely or even selection of cells that express immortality may reduce or avoid the need for new samples (Stephens et al., 2018). Nevertheless, the welfare of animals involved must be considered (Croney et al., 2018). As the number of animals demanded will be only a fraction of that required for slaughter-based meat production, the animals providing cells will probably be kept at higher welfare standards, as measured by accepted assessment protocols (Welfare Quality®, 2009a,b,c) because of their extremely reduced numbers and their high value to the industry. As for the welfare of animals in the remaining conventional meat production in the year 2040, we present the total number of farm animals per main species and their position in terms of animal welfare, in the unlikely case of all meat being produced through conventional processes (Figure 2A), and we discuss two main scenarios for the year 2040 (Figure 2): (A) average farm animal welfare decreases due to a pressure for low-cost conventional meat and (B) average farm animal welfare increases due to a niche-market developing for traditional meat, and a consequent demand for high quality meat, including the addressing of environmental and animal welfare concerns.

The first scenario (Figure 2A) simulates the average total number of cattle, pigs, and chickens involved in farm production in the year 2040 and the welfare of each species. The second scenario (Figure 2B) represents a reduction of 60% of animal use in meat production with a decrease in the average welfare of the remaining farm animals, due to a potential increase in economic pressure. Although cell-based meat is still very expensive and consequently generates high-cost products (Stephens et al., 2018), future large-scale plants and continuous cultivation of cells are expected to considerably reduce the price (Specht et al., 2018). Assuming that in the year 2040, cell-based meat will be widely accessible, and there may be a pressure for the remaining slaughter-based meat production to be at lower cost, to compete with the cell-based products. In this case, average farm animal welfare may decrease due to the increased market pressure for intensive cost-effective production. Hence, although the total size of slaughter-based meat production will be smaller, its proportional impact may be worse, both in relation to animal welfare, environmental issues, and public health matters, including increased disease risks (e.g., *Salmonella* and *Campylobacter*) and greater use of intensively-farmed land to provide the necessary feed (Tubb and Seba, 2019). In this context, the current grains and cereals used in animal production will still require extensive land (Steinfeld et al., 2006) even though they are directly edible by humans (Leitzmann, 2014; FAO, 2018c). This renders conventional meat from grain-based diets intrinsically inefficient in terms of reducing human hunger in the world. The projection for growth in cropped land use is colossal, reaching 3 billion tons of cereals in 2050 (FAO, 2012), in a scenario where alternative meats were not considered. In addition, the animal production sector has been engaged to improve feed conversion so that it is more efficient (Steinfeld et al., 2006), which may result in additional animal welfare problems. One last reason that may force a negative impact of cell-based meat establishment on animal welfare is a putative stimulation of higher global meat consumption, independent of origin (cultured or traditional; Stephens et al., 2018), resulting in increased meat demand regardless of production methods.

The third scenario (Figure 2C) represents higher welfare for the remaining farm animals through a dominance of cell-based meat in the market of low-priced meat and, consequently, high quality or niche demand for traditional meat. According to consumer acceptance studies, willingness to both try and regularly consume cell-based meats is related to its perceived positive impact on animal welfare and environment (Laestadius and Caldwell, 2015; Wilks and Phillips, 2017; Mancini and Antonioli, 2019; Valente et al., 2019), but lower costs for this product may also enhance its consumption (Gaydhane et al., 2018). Therefore, conventional meat may become more expensive, segmented as a luxury food (Post, 2012). Such products are frequently branded and labeled as green, environment and animal-friendly, and consumers are likely to pay premium prices for those attributes (Orsato, 2009) which, in turn, lead to production systems improvements. This may, consequently, allow for higher animal welfare on the remaining conventional farms. Reasons for higher welfare in this case are related to a greater possibility for the adoption of alternative systems for conventional meat production, such as those using free-range pigs and broiler chickens. Outdoor raising systems for pigs generally improve their health and behavior, since animals enjoy more space, access to natural resources, and social contact. It also improves pigs’ mothering and reproductive ability, reduces piglet mortality and the number of pigs with poor leg conditions (Gourdine et al., 2010), as well as increases in social-play and decreased conflict behavior and stereotypies (Nakamura et al., 2011). However, it will still require improvements in pig growth rates (Park et al., 2017) if it needs to compete with confined systems as a low-cost production method. Thus, if traditional pork achieves higher prices as a consequence of cell-based pork availability, the pressure to reduce costs may decline. Likewise, free-range broiler chickens raised in open fields can enjoy improvements in their physical activities and behavioral diversity (El-Deek and El-Sabrout, 2019). Also, animal welfare assessment in free-range systems demonstrates better health and ambience, behavior and psychologic states, less pododermatitis and lameness, an absence of panting, increasing wing-flapping, and prevalence of positive emotional states (Sans et al., 2014). Chickens have been genetically selected for outdoor systems using the so-called “slow growth” lines, which automatically confer higher production costs for the fundamental characteristic of these animals: They grow slower. Using slow growth lines takes roughly double the time and other resources per kilogram of meat produced.

The most significant influence in terms of global animal welfare is, by far, the major reduction in the total number of individual animals involved in food production (Figure 2). This global decrease is in the order of hundreds of millions fewer cattle and pigs and of tens of billions fewer chickens per year. At this point, it is again important to consider the low precision of these calculations but their robustness in order of effects. In other words, even if future reality is 20 or 30% different than the assumptions accepted for our estimations, changes will be highly significant.

For the conventional animal food production that remains, further consideration is needed to understand which systems, either high, low, or intermediate welfare, will be retained and thus define the impact of the innovation on the average welfare of the remaining farm animals. It is likely that further development in farm animal welfare regulations and animal protection laws will remain important. In addition, a stronger focus on welfare regulations for wild animals is likely required in many jurisdictions, to ensure that the outcome of substitution of farm animals by wild animals is associated with less overall suffering and that no increase in human activities that cause wild animal suffering will be allowed. Additionally, it seems possible to foresee potential changes in the human-animal relationships when meat production is uncoupled from animal raising and slaughter, with the mitigation of relevant barriers to animal protection and a recognition of animals as subjects by legislation.

#### Impact on the Human-Animal Relationship

Eating animal meat sets inconsistencies in the human-animal relationships, as most people consider themselves animal lovers but, at the same time, they are causing suffering in non-human animals (Joy, 2005). In addition, meat eating tends to lead people to withdraw moral concern (Loughnan et al., 2010). It has further been postulated that the institution of animal slaughter constitutes the basis of an implicit right to be violent, which may even be linked to a culture, where violence has a valued place (Burgat, 2017). If these views have validity, the development of meat which is uncoupled from slaughter will change human-animal relationships in a profound way.

Animal-based products often have had their names changed to create distance from their animal origin (e.g., beef and pork as opposed to cattle and pigs). Historically, the division between words for animals and their meat emerged because of the French-speaking nobility eating the meat of the animals raised by English-speaking workers (Quinley and Mühlenbernd, 2012). This cultural dissociation of conventional meat products from the animals from which they originate has increased recently, separating killing an animal to produce food from the stages of purchasing, distribution, preparation, and consumption (Buscemi, 2014). The divergent nomenclature is related to the concept of the absent referent, which is anything whose original meaning is undercut as it is absorbed into a different hierarchy of meaning; in this case, the original meaning of animals’ fates is absorbed into a human-centered hierarchy (Adams, 2000). Even though references to the connection between animal and meat were reduced, many people still experience cognitive dissonance whenever something reminds them of the animal origin of meat (Harmon-Jones et al., 2009), which then evokes the meat paradox. To reduce the moral burden, people often minimize harm, deny responsibilities, and diffuse the identity implications of their acts (Bastian and Loughnan, 2017). Thus, as meat is detached from being raised under low welfare conditions and the killing of animals, this moral discomfort should disappear, allowing for unrestricted defense of animal welfare and animal life. This new freedom, in turn, may allow for the recognition that animals are morally relevant individuals, in other words, that they are subjects of a valuable life. Although a simple solution for these moral ambiguities is to follow a plant-based diet, meat consumption is strongly established into most global societies. Carnism is the ideology of meat consumption, where people, as omnivores, choose to eat meat even without the necessity of doing so (Joy, 2011). In this context, Monteiro et al. (2017) discuss two types of carnism: carnistic defense and domination. The first one relates to the meat paradox, supporting eating meat and denying animal suffering in the context of meat production. The carnistic domination is based on the hierarchy between humans and animals, justifying killing animals for human purposes and endorsing human superiority.

Independently of carnism type, the justification of killing animals to produce meat, which is a highly valued human food, may impair improvement of many areas of animal protection. The industrial meat production in typical western urban societies is associated with normalization of animals as having only instrumental value, and with killing animals. Thus, against this background, difficulties arise in recognizing the intrinsic value of individual animals and their rights to integrity and dignity. A right to integrity may be challenged by cell-based meat, confronting virtue ethics, which strives for excellence in character (Hursthouse, 2011) and deontological theory. In modern society, it becomes natural and somewhat necessary to treat animals as resources. This may relate to a generalization, which resides in the banalization of evil (Arendt, 1963). For instance, Giedion (1948) described as follows, the serial killing of animals in slaughterhouses: “What is truly startling in this mass transition from life to death is the complete neutrality of the act. One does not experience, one does not feel; one merely observes.” Indeed, meat is, perhaps most of all, a relationship with animals that is essentially about killing (Burgat, 2017). Therefore, the processes related to meat production may be characterized as a type of desensitization in people (Schacter et al., 2011), because the exposure to dreadful experiences routinely may reduce emotional responsiveness.

If the expectations of price, taste, and appearance of meat can be achieved by cell-based meat, consumers may accept it as a regular food (Bryant and Barnett, 2018). Also, there is strong evidence of cell-based meat consumer acceptance because of its welfare benefits (Laestadius and Caldwell, 2015; Wilks and Phillips, 2017; Mancini and Antonioli, 2019; Valente et al., 2019). In addition, when potential consumers are further informed about environmental or animal welfare benefits − which improves their awareness about those benefits – their willingness to consume increases (Verbeke et al., 2015; Bekker et al., 2017; Weinrich et al., 2019). Thus, since willingness-to-pay regarding animal welfare is related to a social consensus that it has a moral value (Bennett and Blaney, 2002), knowledge about the positive impacts on animals provided by alternative meat production may result in an important contribution to the establishment of this product in the market. Therefore, besides the positive implications of cell-based meat for animals, there may be indirect animal ethics gains in terms of freedom to consider animals as an end in themselves.

**Figure 3:**
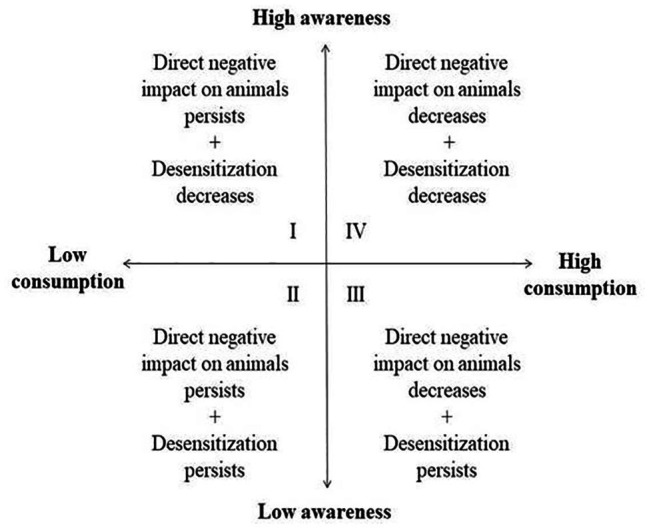
Direct consequences to animals and indirect effects on animal ethics of different levels of cell-based meat consumption and awareness of its animal ethics consequences.

In Figure 3, we represent a possible relationship between the consumption of cell-based meat and the awareness of its consequences in improving animal ethics issues. We projected four different contexts, which are represented anticlockwise from left to right: (1) low consumption of cell-based meat and high awareness (quadrant I) may maintain a direct negative impact on animals but may decrease the desensitization; (2) low consumption and low awareness (quadrant II) may also have a persistent direct negative impact on animals and continued desensitization; (3) quadrant III, with high consumption and low awareness, shows the direct negative impact on animals that may decrease, but the desensitization may persist; and (4) finally, quadrant IV presents high consumption of cell-based meat and high awareness, which may decrease both the direct impact on animals and desensitization.

As meat has traditionally required major animal inputs, resulting in significant impacts on their lives, from being selectively bred to being killed (Mouat et al., 2019), in addition to being closely confined, the consumption of cell-based meat may be a new determinant of animals’ interests and the quality of their lives. Growing awareness, despite urbanization, of the practices of animal production has had an important impact on the ethics of what we eat (Mouat et al., 2019). Phillips (2015) has argued that it is not relative welfare that matters to animals, and therefore to us, but the absolute number of animals that are suffering worldwide. This is further argued by Phillips (2015) to be increasing, because more animal production uses small animals, so more are eaten; more are grown in developing countries without welfare standards and in intensive production systems (Reis and Molento, 2019); and demand for meat is increasing worldwide. While the major switch from slaughter-based to cell-based and plant-based meat consumptions will directly reduce farm animal suffering (quadrants III and IV), the animal ethics improvements will likely depend on decreasing the banalization of animal suffering (Singer, 1995), i.e., decreasing the present levels of desensitization regarding animals (quadrants I and IV). The important direct gains to animals from the decision to buy alternative meats, even when based on non-animal related reasons such as price or human health issues (quadrant III), deserve proper recognition, since from an animal point of view, what matters is not what we think or feel, but what we actually do (Webster, 2016). This recognition does not exclude the importance of striving for decreased desensitization, since this is essential if broader and more permanent gains for animal welfare are to be achieved. In other words, the improvement of the relationship between human and non-human animals in a broad sense seems to be dependent on increasing both the consumption of alternatives to conventional meat and the levels of awareness regarding the role of alternative meats in uncoupling meat from animal suffering and slaughter (quadrant IV).

Our hypothesis is that alternative meats may diminish desensitization toward animals, since people will not have to tolerate the necessary animal suffering and killing for the sake of meat consumption. From a broader perspective, the concepts of animal rights and animals as subjects-of-a-life (Regan, 2004) may find more overall support when meat production is uncoupled from the need to kill animals. However, this may require specific actions to increase awareness of animal ethics issues, since other factors may lead the transition to alternative meats. Thus, even though the transition from traditional meat to cell-based meat will have an intrinsic direct positive impact on farm animals, the promotion of awareness may increase the human-animal relationships in a more generalized sense.

## Conclusions

The development of a slaughter-free meat chain will have significant practical and animal ethics impacts on our relationship with non-human animals, which are wider than the *prima facie* benefits to farm animals. This is supported by utilitarian, deontological, and virtue ethical principles applied to animals. Considering the many uncertainties involved, especially those regarding the rate of substitution, which is dependent on acceptance levels of alternative meats by different societies, the resolution of technological challenges, and the need for transparency to avoid significant drawbacks, it is highly likely that a major disruptive change is on the horizon. Gains in environmental resources such as land, water, and biomass are likely to be very significant, while energy costs per kilogram may remain high for cell-based meat. More research is needed to understand the consequences of new meat alternatives for the welfare of the remaining farm animals, since it will depend on economic pressures and the strategies that will be adopted by the conventional meat chain. Finally, alternative meats may diminish desensitization toward animals, since people will not have to allow for some kind of necessary animal sufferings for the sake of meat consumption. Thus, there may be indirect animal ethics gains in terms of freedom to consider animals as an end in themselves. Our relationships with non-human animals may be about to change to a more respectful, mutualistic relationship, for the benefits of all concerned.

## Author Contributions

MH contributed to conceptualization, investigation, methodology, writing, review, and editing. CM contributed to project administration, supervision, conceptualization, investigation, methodology, writing, review, and editing. GR and CP contributed to conceptualization, writing, review, and editing. All authors contributed to the article and approved the submitted version.

### Conflict of Interest

The authors declare that the research was conducted in the absence of any commercial or financial relationships that could be construed as a potential conflict of interest.
